# Chiu Weifan—A founder of plant virology in China and the pioneer of agricultural science

**DOI:** 10.1093/procel/pwaf040

**Published:** 2025-05-18

**Authors:** Huan Liu, Qiangyu Xiang, Zongzhen Wu, Hao Cheng

**Affiliations:** Department of History of Science and Scientific Archaeology, University of Science and Technology of China, Hefei 230026, China; State Key Laboratory of Virology, Wuhan 430072, China; Department of History of Science and Scientific Archaeology, University of Science and Technology of China, Hefei 230026, China; Department of History of Science and Scientific Archaeology, University of Science and Technology of China, Hefei 230026, China; Institute of Microbiology, Chinese Academy of Sciences, Beijing 100101, China

In the hallways of the University of Wisconsin–Madison, a portrait of a Chinese scientist hangs prominently. Likewise, at the historical exhibition of China Agricultural University, another portrait can be found, accompanied by the inscription: “Plant Pathologist, Plant Virologist, Mycologist, Agricultural Educator.” This individual is none other than Professor Chiu Weifan (裘维蕃, 1912–2000) (Fig. 1), a seminal figure in the establishment of plant pathology and virology in China. His pioneering research significantly advanced both the prevention and control of plant diseases and the broader development of plant virology in the country. Professor Chiu was an academician of the Chinese Academy of Sciences (CAS) and held several esteemed positions, including Professor at Beijing Agricultural University and China Agricultural University, one of China’s first doctoral advisors, Vice President of the China Association for Science and Technology, President of the Chinese Society for Plant Pathology, and President of the Mycological Society of China. In recognition of his outstanding contributions, he received numerous prestigious accolades, such as the National Science and Technology Progress Award (Third Class) and the National Natural Science Award (Third Class). Additionally, he was listed in *The International Who*’*s Who in Asia and Australasia*, *The International Who*’*s Who*, and *Five Thousand Personalities of the World*. Among his many achievements, Professor Chiu pioneered the artificial cultivation of edible fungi and was among the earliest scientists to observe the heterokaryotic phenomenon in fungal hyphae (Chiu and Walker, 1949). On the international stage, he was the first to propose a quantitative formula for disease severity assessment (Chiu et al., 1949), which has since become widely adopted as the disease index (Liu and Chiu, 1995). His work on controlling major diseases of Chinese cabbage—clubroot, downy mildew, and soft rot—provided essential solutions for disease management. As early as the 1960s, his research demonstrated that wheat dwarf disease in China was caused by a virus transmitted by the gray planthopper, laying the foundation for future disease prevention efforts (Cai et al., 1979). In the 1980s, Professor Chiu proposed the theory of induced plant resistance to viruses and successfully developed “NS-83,” a resistance-inducing agent (Lei et al., 1984), which proved effective in suppressing viral diseases in crops such as tomatoes and tobacco (Lei et al., 1987). His pioneering work not only safeguarded agricultural production but also had a profound impact on the advancement of related scientific disciplines in China.

**Figure 1.**
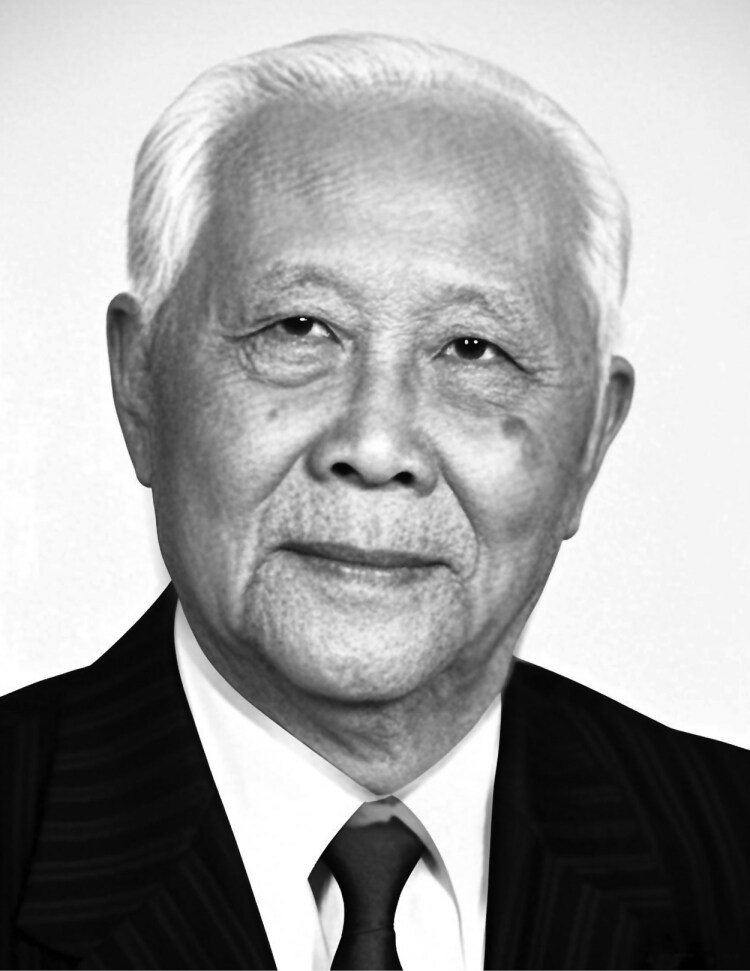
Professor Chiu Weifan (1912–2000).

Professor Chiu Weifan was born on May 15th, 1912, in Wuxi, Jiangsu Province, into a scholarly family, developing an early interest in literature and natural sciences. In 1931, he entered Jinling University (金陵大学), studying plant pathology under eminent scholars Dai Fanglan (戴芳澜) and Yu Dafu (俞大绂). After graduation in 1935, he remained there as a faculty specializing in plant diseases. In 1940, at Fujian Agricultural College, he conducted extensive field research, publishing influential findings including *A Report on Plant Diseases of Economic Crops in Fujian*. In 1944, Chiu pursued doctoral studies at the University of Wisconsin–Madison under renowned plant pathologist J.C. Walker, earning his Ph.D. in 1947 with research on cucurbit black rot (Fig. 2). During this period, he was inducted into Sigma Xi Scientific Research Honor Society. Despite favorable research opportunities abroad, he resolutely returned to China, dedicating himself to national agricultural development. He held faculty positions at Tsinghua University and Beijing Agricultural University, significantly contributing to research, teaching, and mentoring. Many of his students became leading figures in plant pathology, virology, and mycology, continuing his academic legacy.

**Figure 2.**
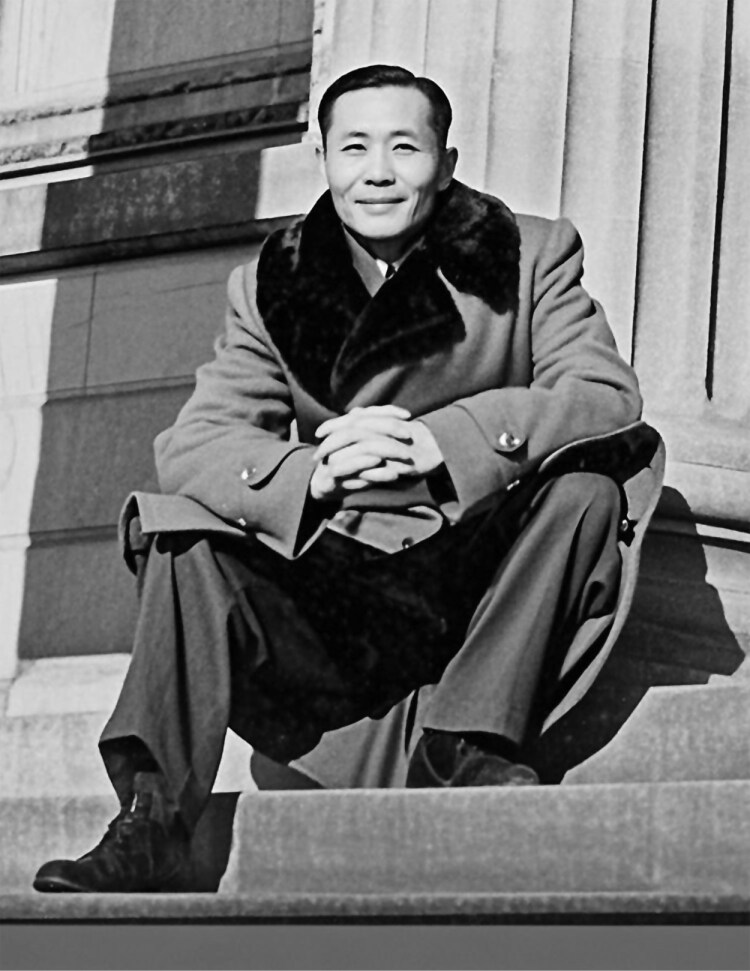
Professor Chiu Weifan at the University of Wisconsin–Madison, 1947.

## Pioneering the artificial cultivation of edible mushrooms

In the mid-1930s, edible mushroom cultivation in China was limited, primarily serving Western cuisine in cities like Shanghai. Recognizing its untapped potential, mycologist Chiu Weifan extensively investigated traditional methods across regions including Mount Huang (Anhui), Daba Mountains and Mount Emei (Sichuan), and Liuzhou (Guangxi). He documented techniques for shiitake, white fungus, and wood ear fungus, classified straw mushrooms, and isolated multiple pure fungal strains. Building upon these findings, Chiu developed an innovative artificial cultivation protocol. His approach involved enriching sterilized sawdust substrates with selected nutrients and inoculating them with pure fungal strains, achieving China’s first successful underground cultivation of golden needle mushrooms (*Flammulina velutipes*). This breakthrough enabled controlled, scalable mushroom farming, replacing unpredictable natural inoculation.

Despite the disruptions of the late 1930s and early 1940s, Chiu’s pioneering research laid a critical scientific foundation for China’s mushroom industry. In 1941, invited by Professor Dai Fanglan (戴芳澜), Chiu shifted his research to Agaricales and Boletales fungi, utilizing Yunnan’s rich botanical resources and publishing influential studies. Chiu’s landmark monograph, *Chinese Edible Mushrooms and Their Cultivation*, was China’s first comprehensive guide on mushroom cultivation. His subsequent work, *Illustrated Monograph of Boletes in Yunnan* (1957), significantly advanced fungal taxonomy. Internationally recognized as one of the “Seven Leading Experts in Global Mushroom Taxonomy,” Chiu’s research was incorporated into the authoritative *Encyclopedia of Mycology*, demonstrating his lasting global impact.

## Discovery of the heterokaryotic phenomenon in fungal hyphae

The heterokaryotic phenomenon describes the coexistence of genetically distinct nuclei within a single fungal hyphal cell, occurring after gamete fusion but before nuclear fusion (*karyogamy*). This condition significantly enhances fungal genetic diversity, adaptability, and evolutionary success. In 1947, Chiu Weifan’s doctoral dissertation at the University of Wisconsin presented pioneering research on fungal heterokaryosis, later published as two seminal articles: *Morphology and Variation of the Cucumber Black Rot Fungus* (Chiu and Walker, 1949) and *Physiology and Pathogenicity of the Cucumber Black Rot Fungus* (Chiu and Walker, 1949). Reflecting later, Chiu noted that this dissertation represented one of the earliest systematic explorations of heterokaryosis in fungal hyphae.

Building upon this foundational work, in 1999, Chiu’s team investigated genetic diversity among natural populations of shiitake mushrooms (*Lentinula edodes*) in a *Fagus longipetiolata* forest in Shaanxi Province. Utilizing fungal heterokaryotic properties, they identified a clear correlation between increased genetic variability and broader geographic distribution (Chiu et al., 1999). These findings validated Chiu’s earlier insights and underscored the practical importance of heterokaryosis for understanding fungal biodiversity, ecology, and evolution.

## Propose a disease severity formula

Accurately assessing plant disease severity is crucial for epidemiological studies and disease control. Traditional methods primarily relied on disease incidence percentages, which simply measured the proportion of infected plants without distinguishing between mild and severe infections. Recognizing the limitations of this approach, Professor Chiu Weifan developed a novel quantitative formula to evaluate disease severity more precisely. During his 1946 doctoral research, Chiu identified a critical flaw in conventional assessment methods while investigating black rot in cucumbers. These methods failed to account for variations in disease severity, providing incomplete epidemiological data. To address this issue, he proposed a disease severity index, which categorized infected plants into different severity levels (e.g., 0, 1, 2, …, N). By using a weighted formula, he derived a standardized index ranging from 0 to 100, ensuring a more precise and comparable measure of disease impact. This groundbreaking disease severity formula was published in the *Journal of Agricultural Research* (Chiu and Walker, 1949) , preceding similar work by British scientists for 2 years. Today, Chiu’s formula remains widely used in plant disease epidemiology, efficacy evaluations of control measures, and other agricultural research areas, demonstrating its lasting impact on the field.

## Control measures for cabbage diseases of clubroot, downy mildew, and soft rot in China

In the early 1950s, three major diseases—clubroot, downy mildew, and soft rot severely threatened cabbage production in China (Chiu, 1960). Professor Chiu Weifan led pioneering research to identify their causes and develop effective control strategies, shaping integrated disease management in Chinese agriculture. Initially, clubroot was thought to result from drought or nutrient deficiencies. However, in 1953, Chiu’s aphid-mediated virus transmission experiments disproved this, identifying Turnip Mosaic Virus as the primary cause, shifting management from irrigation to vector control (Chiu and Liang, 1963). For downy mildew, Chiu’s team conducted field surveys in Beijing, Tianjin, and northeast China, discovering that *Hyaloperonospora brassicae* oospores in soil and plant debris were key inoculum sources. This led to disease-free seedling strategies (Zhang et al., 1963). Soft rot, caused by *Erwinia aroideae* and *Erwinia carotovora*, was linked to pest-inflicted wounds. Chiu’s research emphasized pest control over chemical bactericides, identifying *Phyllotreta striolata* and *Pieris rapae* as key vectors (Chiu, 1955). These innovations significantly improved cabbage disease management, boosting yields and economic returns.

## Prove a virus transmitted by leafhoppers causes wheat dwarf disease in China

Wheat dwarf disease (WDD) severely impacts wheat crops, causing stunting, excessive tillering, leaf chlorosis, and yield losses. Infected plants develop undersized spikes with sparse or no grains, leading to devastating harvest reductions. Professor Chiu Weifan first observed WDD near Shijiazhuang, China. Initially sporadic, the disease escalated into widespread outbreaks.

Chiu systematically studied its symptoms, severity grading, and yield impact (Chiu, 1965). He classified disease severity into four levels: 0 (healthy), 1 (mild), 2 (moderate), and 3 (severe), correlating with yield losses of 52.6%, 66.7%, and 93.2%, respectively (Chiu et al., 1979). Seeking the cause, Chiu’s team conducted virus-free insect transmission experiments, confirming *Psammotettix striatus* (gray leafhoppers) as the primary vector. They identified WDV, related to Japan’s Northern Cereal Mosaic Virus, as the main causal agent (Yang et al., 1979). Some wheat plants also harbored Maize Rough Dwarf Virus (MRDV), leading to blue dwarf symptoms when MRDV predominated.

## Development of “NS-83 virus resistance inducer”

**Figure 3.**
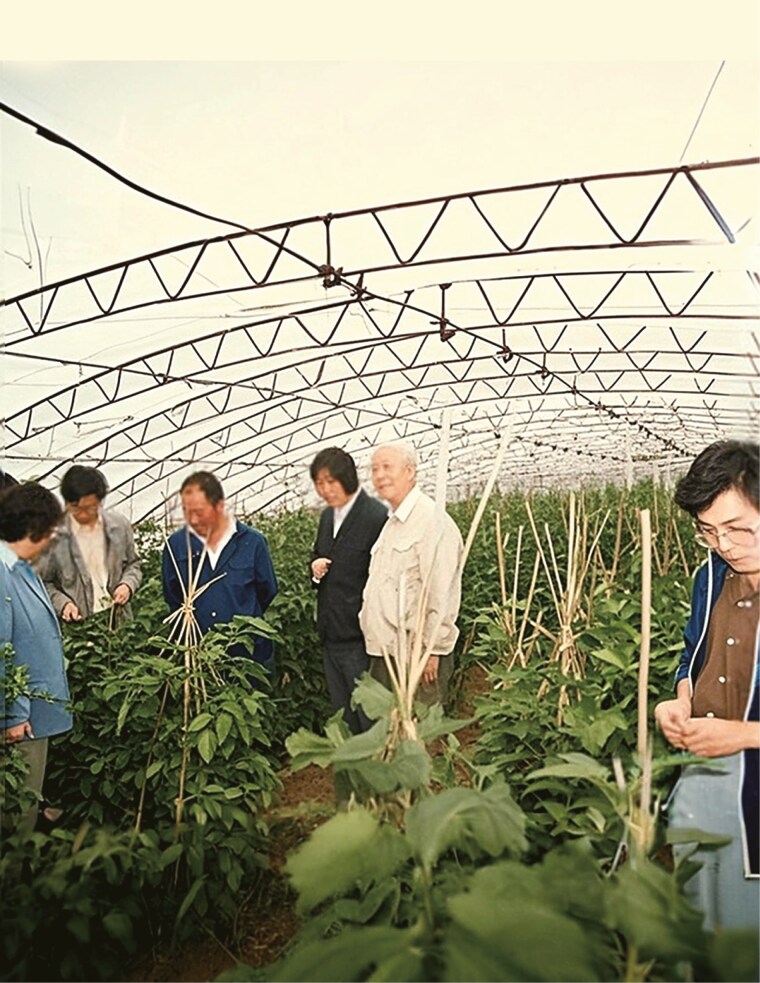
In 1985, Professor Chiu Weifan personally visited experimental fields to investigate the progress of the virus resistance inducer trials.

In the 1980s, Professor Chiu Weifan identified viral diseases as a major threat to Chinese agriculture. With limited antiviral options available, he proposed a novel strategy: inducing plant resistance to viral infections. Under China’s Sixth Five-Year Plan, Chiu led a research team focused on tomato virus diseases. After several years of rigorous experimentation (Fig. 3), they developed NS-83, a virus-resistance inducer that significantly reduced disease incidence and enhanced crop yields by approximately 10% (Liang and Chiu, 1983). Field trials in Tianjin and other regions demonstrated the practical effectiveness of NS-83, leading to its official recognition as a major scientific advancement. In 1991, the project was awarded the First-Class Science and Technology Progress Award (Theoretical Category) by the Ministry of Education and the Third-Class National Natural Science Award. The successful development of NS-83 marked a paradigm shift in plant virus management, paving the way for novel resistance-inducing strategies in agricultural biotechnology.

## Contributing to the nation, serving the agricultural education

**Figure 4.**
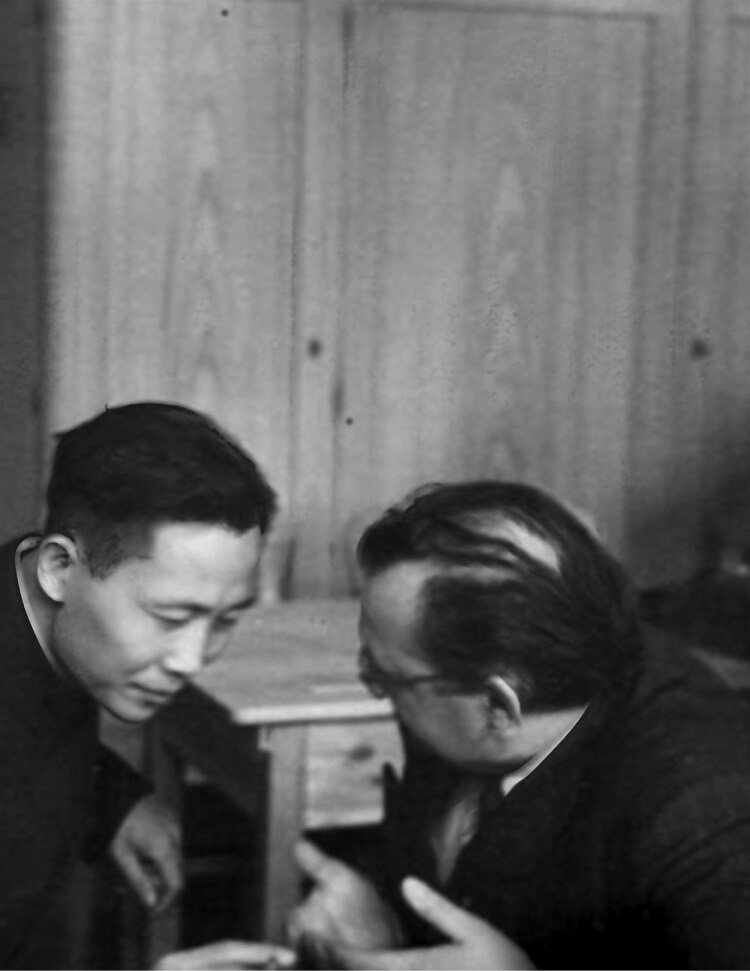
From December 1952 to March 1953, Professor Chiu Weifan participated in the countering bacterial warfare exhibition expert group in Leipzig, Germany, where he engaged in discussions with international experts on the topic.

**Figure 5.**
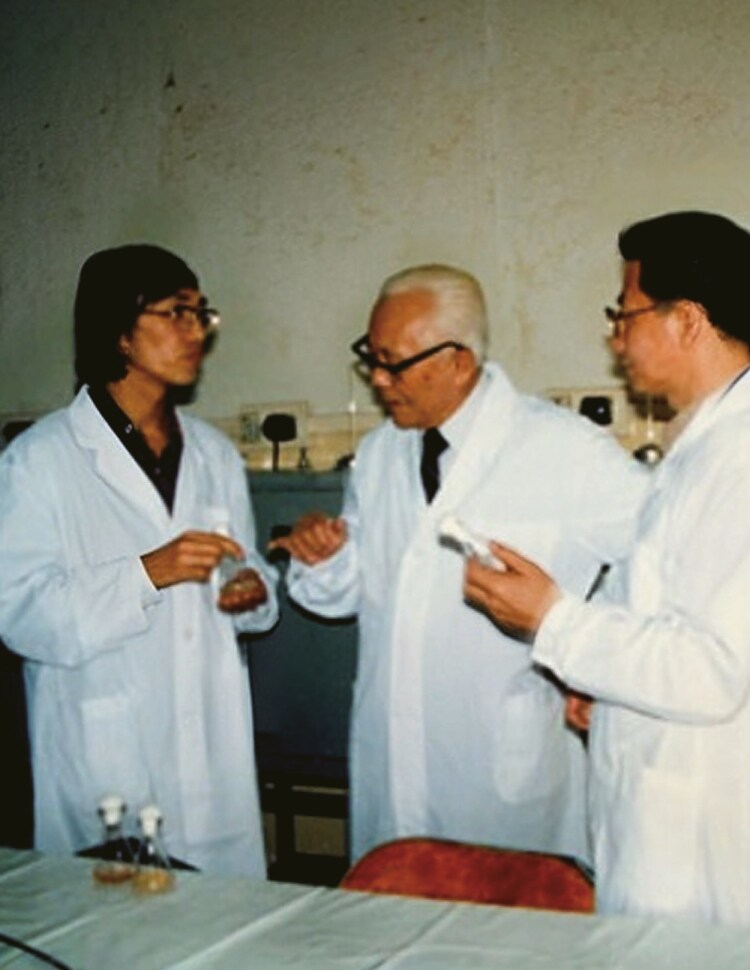
In June 1988, Professor Chiu Weifan (middle) provided guidance to his doctoral students in the laboratory.

Beyond his groundbreaking research, Professor Chiu Weifan was a dedicated educator and science advocate. During the early 1950s, when allegations of biological warfare emerged, Chiu utilized his expertise in microbiology to assist in scientific countermeasures (Fig. 4). He compiled a comprehensive report on American biological warfare research, which he submitted to Vice Premier Lu Dingyi (陆定一), providing crucial evidence for China’s national defense efforts. As a professor at Beijing Agricultural University, Chiu played a pivotal role in shaping modern plant pathology and virology education in China. He was among the first scientists to mentor doctoral students in agricultural sciences, training over 50 master’s and Ph.D. students (Fig. 5). Many of his protégés later became leaders in plant pathology, virology, and mycology. Recognizing the importance of early science education, Chiu led efforts to strengthen biology curricula in Chinese middle schools. He also served as President of the Chinese Society for Plant Pathology and the Mycological Society of China, significantly influencing the development of plant pathology and mycology research in China. His seminal works, including *The Encyclopedia of Mycology and Plant Virology*, have become foundational references for researchers worldwide. Professor Chiu Weifan passed away on September 18th, 2000, at the age of 88. His life’s work embodied scientific excellence, dedication to education, and unwavering commitment to national progress. His pioneering research, innovative disease control strategies, and contributions to scientific education continue to inspire new generations of agricultural scientists.
